# “Are We Feeding Them Enough?” Micronutrient Deficiency in Children Aged Six Months to Fourteen Years in India

**DOI:** 10.7759/cureus.19405

**Published:** 2021-11-09

**Authors:** Sarayoo R Vaidya, Naqvi Syed Gaggatur, Aliya H Sange, Natasha Srinivas, Mubashira K Sarnaik, Yasaswi Pisipati, Ibrahim Sange

**Affiliations:** 1 Internal Medicine and Pediatrics, M S Ramaiah Medical College, Bengaluru, IND; 2 International Health, Charité – Universitätsmedizin Berlin, Berlin, DEU; 3 Research, K. J. Somaiya Medical College, Mumbai, IND; 4 Research, BGS Global Institute of Medical Sciences, Bengaluru, IND

**Keywords:** pediatric nutrition, public health, nutrition status, nutritional deficiency, micronutrient

## Abstract

In this study, we conducted a systematic literature review of the various micronutrient deficiencies (MNDs) that affect children in India and an examination of whether there is a geographic basis for MNDs.

MNDs are a common problem in the developing world, in particular, among children in South Asia. According to the United Nations Children’s Fund, millions of children suffer from stunted growth, cognitive delays, weakened immunity, and diseases because of MNDs. These physical ailments have several economic, social, and public health implications, and they can severely hamper a country’s growth. This study aims to clarify existing data on this topic and highlight the disparities between children living in urban and rural areas in India.

The Preferred Reporting Items for Systematic Reviews and Meta-Analyses guidelines were followed to conduct this systematic literature review of a total of five studies. Study quality was assessed using appropriate checklists, and the studies strengthened the hypothesis that MNDs are common among children in India. Because the selected studies were heterogeneous, no statistical conclusions are drawn here. However, a central premise is that MNDs in children are prevalent in India and are related to poverty. No link between geographic location and MNDs is established; rather, recommendations are made for further research on the topic.

## Introduction and background

Childhood undernutrition is a major public health concern and is the underlying cause of 3 million deaths per year globally. Undernutrition includes stunting, wasting, and deficiencies of essential vitamins and minerals (micronutrients) [1]. Micronutrients play an essential role in human physiology and immunology, but deficiencies are common in children and can have long-term health consequences. A significant proportion of the world’s poor live in India, as do a significant proportion of the world’s malnourished children, and serious inequalities exist in growth, development, and opportunity. Poor dietary quality and undernutrition coexist with poverty, and poor dietary quality and significant micronutrient deficiencies (MNDs) are associated with poor growth during childhood.

India has made tremendous progress on all fronts since independence including food production. To improve the population’s nutrition and health status, the country has launched a wide range of programs such as Integrated Child Development Services, Mid-Day Meals, the National Iron Plus Initiative, the National Iodine Deficiency Disorders Control Programme, and the National Prophylaxis Programme against Nutritional Blindness due to Vitamin A Deficiency. However, a large portion of children still suffers from malnutrition attributable to micronutrition, and deficiencies of micronutrients such as iodine, iron, and vitamin A impact health to the cost of approximately 0.8-2.5% of India’s gross domestic product.

This study aimed to outline the common MNDs present in children aged six months to fourteen years in India via a systematic literature review of existing studies. The minimum age for inclusion in the study was six months because this is the age when complementary feeding practices are commonly initiated. Complementary feeding is defined as foods introduced to supplement when breast milk alone is no longer sufficient to meet infants’ nutritional requirements. The period of complementary feeding is critical for children’s growth, and incorrect feeding practices make a child vulnerable to malnutrition and MNDs.

The maximum age for the children in this study was 14 years because children at this age are classified as adolescents, not children. Both boys and girls were included in the study, and there was no separate consideration of iron deficiency anemia related to girls’ menstruation. In addition, this study aimed to explore whether there were differences in the prevalence of MNDs. To this end, a control group involving children from urban areas in New Delhi was investigated.

Because of time constraints, the focus of this study was limited to the literature on the most common MNDs. Anemia, the most common micronutrient deficiency, affects 50-60% of preschool children and women, whereas vitamin A deficiency and iodine deficiency disorders have improved over the years. There have been limited studies in India on other MNDs, with researchers focusing on interventional, preventive approaches in antenatal care that did not satisfy the inclusion criteria for this study.

This study aimed to clarify existing data on this topic and highlight the disparities between children living in urban and rural areas in India toward the goal of establishing effective interventions to combat these problems. This target population was chosen because of the economic implications for the future lives of children affected by MNDs. The most widespread MNDs are iron, iodine, folate, vitamin A, and zinc, which are common contributors to poor growth, intellectual impairment, perinatal complications, and increased risk of morbidity and mortality.

Methodology

One principal reviewer was involved in all steps of this systematic literature review. The Preferred Reporting Items for Systematic Reviews and Meta-Analyses (PRISMA) guidelines were employed to conduct this review.

Study Population

The sample of studies for review was restricted to published white literature on deficiencies concerning four common micronutrients, iron, vitamin A, zinc, and iodine. The study population was boys and girls living in rural India aged between six months and fourteen years. Moreover, for comparison, a control population of children residing in urban areas of the region was investigated.

This study was not funded. Given this, the first exclusion criterion was studies for which free full-text was not available. Studies not conducted exclusively in India or those including neonates (children less than one month of age) in the research group were excluded. Studies with adolescents were considered as long as the children were younger than 14 years old.

Search and Study Selection Criteria

To identify articles for the study, the electronic literature databases PubMed and Google Scholar were searched, with dates limited to studies published after 2000 until 2020. The language was restricted to studies originally published in English. The inclusion criteria were as follows: (1) presence of a common MND in a child who had begun eating solid food; (2) restriction to common MNDs with significant public health importance, namely, anemia, vitamin A, zinc, and iodine; (3) studies conducted in India (urban or rural); and (4) narrative literature reviews. Nonsystematic, that is, prospective/retrospective observational studies were excluded.

The database search keywords in Google Scholar included “micronutrient deficiency,” “India,” “children,” “rural nutrition deficiency,” “urban nutrition deficiency,” and “pediatric nutritional deficiency India.” Subsequently, Boolean operators were introduced using PubMed’s advanced search function to make the study parameters more concise, such as the following: ((((((((“micronutrients”[Pharmacological Action] OR “micronutrients”[MeSH Terms]) OR “micronutrients”[All Fields]) OR “micronutriments”[All Fields]) OR “trace elements”[Pharmacological Action]) OR “trace elements”[MeSH Terms]) OR (“trace”[All Fields] AND “elements”[All Fields])) OR “trace elements”[All Fields]) OR “micronutrient”[All Fields]) AND (((((“deficiences”[All Fields] OR “deficiencies”[All Fields]) OR “deficiency”[MeSH Subheading]) OR “deficiency”[All Fields]) OR “deficient”[All Fields]) OR “deficients”[All Fields]) AND (((“india”[MeSH Terms] OR “india”[All Fields]) OR “india s”[All Fields]) OR “indias”[All Fields]) AND ((((((“child”[MeSH Terms] OR “child”[All Fields]) OR “children”[All Fields]) OR “child s”[All Fields]) OR “children s”[All Fields]) OR “childrens”[All Fields]) OR “childs”[All Fields]).

Data Extraction

When a substantial number of articles were identified, free full-text articles were accessed for review. Studies cited within the main texts were also analyzed for suitability based on the inclusion criteria. A few systematic reviews that did not fit the inclusion criteria were also analyzed to guide this review. Texts were then screened according to the PRISMA guidelines.

First, data from each study were extracted and collated. Second, data were assessed using quality markers based on Cochrane reviews. Multiple markers were used that will be detailed later in the article. There was substantial heterogeneity in the searched literature, and thus, a meta-analysis was not indicated. Instead, the studies were compared based on the overall trends in outcomes observed in the different geographic regions(urban or rural). For each outcome, the data were extracted separately for urban versus rural populations.

## Review

A total of 57 studies were identified from the literature search of the online search engines mentioned above [1-56]. Abstracts of individual texts were screened based on the preliminary inclusion criterion of free full-text availability, yielding a total of 32 articles. The full-text articles were then extracted and reviewed in detail based on the specific inclusion criteria. Although a total of 11 articles fit the criteria loosely, three were excluded as they did not meet the inclusion criteria of the study [33,34,55].

**Figure 1:**
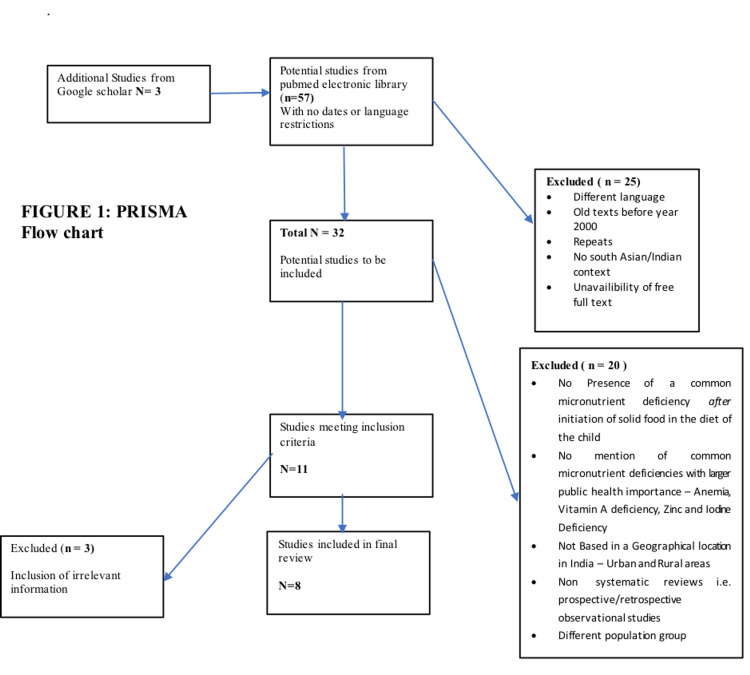
PRISMA flowchart. PRISMA: Preferred Reporting Items for Systematic Reviews and Meta-Analyses

Overall, eight articles [4,8,10-16,56] satisfied the inclusion criteria, and five were selected for inclusion in this systematic review. The studies included for the final review and analysis were systematic literature reviews. All studies were conducted in India, and children were the main study populations in all studies. Figure 1 presents the PRISMA flow diagram of study selection for this review.

Gonmei and Toteja and Srihari et al. presented a general overview of micronutrients that helped provide a basis for comparing the different MNDs [4,56]. Further, the studies by Rakesh, Yadav et al., and Vijayaraghavan, addressed anemia, iodine, and vitamin A deficiency in rural communities, respectively [10,11,15]. These three studies were also chosen because they were published close to each other, although their study populations were different, resulting in high heterogeneity was achieved. However, a basis remained for comparison across studies (Table 1).

**Table 1:** Characteristics of included studies.

Study	Country	Study design	Outcome (+/−) significant micronutrient deficiency	Age group	Geographic region	Subcategories
Gonmei and Toteja, 2018 [4]	India	Narrative review	+	Infants, preschool children, adolescents	Urban and rural	Anemia, zinc, and vitamin A deficiency
Srihari et al., 2007 [56]	India	Systematic review	+	School-going children	Urban	Anemia, zinc, and vitamins A, B, C, and D deficiency
Rakesh, 2017 [15]	India	Systematic review	+	Children, adolescents, women	Urban	Anemia
Yadav et al., 2018 [11]	India	Narrative review	−	Children, women	Rural	Iodine deficiency
Vijayaraghavan, 2018 [10]	India	Narrative review	−	Children	Rural	Vitamin A deficiency

Gonmei and Toteja [4] analyzed existing studies on anemia, zinc, iodine, and vitamin A deficiencies across multiple age groups, although they primarily extracted data on school-going children. Moreover, they presented a basic overview of MND trends across multiple school districts and did not compare urban and rural populations. Their findings suggested that deficiency is more prevalent among children in rural areas because of poverty. Overall, Gonmei and Toteja concluded that MNDs, particularly iron, are a major health problem in the country, but vitamin A deficiency and iodine deficiency disorders have improved [4].

Srihari et al. [56] provided a perspective of MNDs among school-going children from middle- and higher-income families in urban areas. Interestingly, they identified MNDs as being associated with obesity and diets containing processed foods. Specifically, they concluded that attention is warranted on children from middle- and higher-income families, especially with respect to the high prevalence of anemia, overweight, and obesity. In addition, they reported that there are indications that MNDs exist, but sufficient data are lacking, in particular, biochemical data [56].

The other three studies were analyses of individual MNDs. The study conducted by Rakesh [15] reported the following: prevalence of anemia among adolescents according to recent studies is approximately 30%, and prevalence of severe anemia is less than 1% in all studies. Further, anemia among tribal women and children ranged from 78.3% to 96.5% [15]. Separately, Yadav et al. [11] and Vijayaraghavan [10] addressed the prevalence of deficiency by examining the national intervention programs for iodine and vitamin A deficiency, respectively. Yadav et al. [11] collected insufficient data for establishing current iodine deficiency among children because of the confounding effect of maternal iodine deficiency as a contributor to low stores in children [11]. Vijayaraghavan [10] identified significant vitamin A deficiencies in rural children as opposed to children in urban areas by examining the prevalence of children with preventable blindness in both groups.

The conclusions from all three studies strengthened the hypothesis of this study that MNDs are more prevalent in rural areas. The 10-item Critical Appraisal Skills Program checklist was used to analyze the quality of the five studies (Table 2). All studies achieved scores of at least 80% of checklist criteria met.

**Table 2:** CASP checklist of five studies. CASP: Critical Appraisal Skills Program

Questions	Gonmei and Toteja, 2018 [4]	Srihari et al., 2007 [56]	Rakesh, 2017 [15]	Yadav et al., 2018 [11]	Vijayaraghavan, 2018 [10]
Did the review address a clearly focused question?	Could not tell	Yes	Yes	Yes	No
Did the authors look for the right type of papers?	Yes	Yes	Yes	Yes	No
Do you think all the important, relevant studies were included?	Yes	No	Yes	Yes	Yes
Did the authors do enough to assess the quality of the included studies?	Yes	Yes	Yes	Yes	Yes
If the results of the review have been combined, was it reasonable to do so?	Yes	Yes	Yes	Yes	Yes
What are the overall results of the review?	Yes, micronutrient deficiency is a problem in the population	No clear problem defined and no causal analysis	Yes, the prevalence of the problem was clear with odds ratios	Could not tell	Yes, the prevalence of the problem was clear with odds ratios
How precise are the results?	Precise: population sample is large	Precise	Precise	Precise	Precise
Can the results be applied to the local population?	Yes	Yes	Yes	Yes	Yes
Were all important outcomes considered?	Yes	Yes	No	Yes	Yes
Are the benefits worth the harms and costs?	Yes	Yes	Yes	Yes	Yes
Number of criteria met out of 10 (%)	9 (90%)	8 (80%)	9 (90%)	9 (90%)	8 (80%)

The authors of the studies analyzed in this review identified different MNDs in children, concluding that poverty was the underlying cause of the deficiencies irrespective of their type. The studies selected for this review were of good quality and fit the inclusion criteria. However, despite this review, it is still not possible to provide a clear answer to the research question of whether the geographic location has any impact on MNDs in children. Research that produces clear empirical findings will require proper comparison and control groups with supportive data to establish clear relationships, for instance, between children from families of low and high socioeconomic status living in the same geographic area.

There are some limitations of this study that affect the interpretations of the data. First, because there was limited time to conduct the review with only one reviewer, there is likely some bias in the results. Particularly, studies that were more easily accessible were selected over those that required author permission or were going to take a long time to extract data. Second, it was not possible to study the combined effects of numerous MNDs within one individual. For instance, vitamin B and C deficiencies can coexist with anemia, making it difficult to determine which, if any, deficiency is accounting for a child’s condition. A larger analysis will help establish the manifestations of deficiency. Third, there were insufficient data available regarding gender disparity in MNDs, and thus, future researchers should investigate differences in these deficiencies between boys and girls in the same location or those belonging to the same socioeconomic status. Future researchers can also explore trends in childhood obesity and MNDs in urban populations and in certain endemic areas such as India’s Pacific Coast.

## Conclusions

This study helped illustrate the common MNDs prevalent among children in India and attempted to examine whether geographic location contributes to this problem. Because of heterogeneity, it was not possible here to reach clear conclusions based on statistics; instead, conclusions had to be drawn from the literature. Because of these limitations, it was not possible to establish a clear causal link, and there remains a range of potential work future researchers can pursue in this area. Overall, however, MNDs in children are a global health problem, and the findings from this study can be applied to other low- and middle-income countries.
